# *cc*SOL *omics*: a webserver for solubility prediction of endogenous and heterologous expression in *Escherichia coli*

**DOI:** 10.1093/bioinformatics/btu420

**Published:** 2014-06-01

**Authors:** Federico Agostini, Davide Cirillo, Carmen Maria Livi, Riccardo Delli Ponti, Gian Gaetano Tartaglia

**Affiliations:** ^1^Gene Function and Evolution, Bioinformatics and Genomics, Centre for Genomic Regulation (CRG), 08003 Barcelona, Spain and ^2^Universitat Pompeu Fabra (UPF), 08003 Barcelona, Spain

## Abstract

**Summary:** Here we introduce *cc*SOL *omics*, a webserver for large-scale calculations of protein solubility. Our method allows (i) proteome-wide predictions; (ii) identification of soluble fragments within each sequences; (iii) exhaustive single-point mutation analysis.

**Results:** Using coil/disorder, hydrophobicity, hydrophilicity, β-sheet and α-helix propensities, we built a predictor of protein solubility. Our approach shows an accuracy of 79% on the training set (36 990 Target Track entries). Validation on three independent sets indicates that *ccSOL omics* discriminates soluble and insoluble proteins with an accuracy of 74% on 31 760 proteins sharing <30% sequence similarity.

**Availability and implementation:** *cc*SOL *omics* can be freely accessed on the web at http://s.tartaglialab.com/page/ccsol_group. Documentation and tutorial are available at http://s.tartaglialab.com/static_files/shared/tutorial_ccsol_omics.html.

**Contact:** gian.tartaglia@crg.es

**Supplementary information:** Supplementary data are available at *Bioinformatics* online.

## 1 INTRODUCTION

Algorithms for prediction of protein solubility (Wilkinson and Harrison, 1991) and aggregation (Fernandez–Escamilla *et al.*, 2004) provide a solid basis to investigate physico-chemical determinants of amyloid fibril formation and associated diseases (Conchillo–Solé *et al.*, 2007; Tartaglia *et al.*, 2004). In the past years, an *in vitro* reconstituted translation system allowed the large-scale investigation of *Escherichia coli* proteins solubility (Niwa *et al.*, 2009), thus providing the opportunity for the development of predictive methods such as *cc*SOL (Agostini *et al.*, 2012). In *cc*SOL, coil/disorder, hydrophobicity, hydrophilicity, β-sheet and α-helical propensities are combined together into a solubility propensity score that is useful to investigate protein expression (Baig *et al.*, 2014) as well as bacterial evolution (Warnecke, 2012). Other methods have been developed to predict protein solubility based on amino acid characteristics. For instance, PROSO II (Smialowski *et al.*, 2012) exploits occurrence of monopeptides and dipeptides to estimate heterologous expression in *E.coli*. PROSO II was trained on the pepcDB database [now Target Track (Berman *et al.*, 2009)] that stores target and protocol information provided by Protein Structure Initiative centers. Both *cc*SOL and PROSO II perform accurate predictions when used to respectively predict endogenous or heterologous soluble expressions [*cc*SOL: 76% accuracy; PROSO II: 75% accuracy (Smialowski *et al.*, 2012)]. We found that the experimental status of several Target Track entries (http://sbkb.org/tt/) has been recently updated and new data are available to train predictive methods (see Supplementary Material). Here, we introduce a novel implementation of the *cc*SOL method, called *cc*SOL *omics*, to perform large-scale predictions of endogenous and heterologous expression in *E.coli*. Our algorithm has been trained on non-redundant Target Track entries to identify soluble and insoluble regions within protein sequences. We envisage that *cc*SOL *omics* will be useful for protein engineering studies, as it allows the investigation of sequence variants in large datasets.

## 2 WORKFLOW AND IMPLEMENTATION

The *cc*SOL *omics* server allows the investigation of large protein datasets (see Supplementary Material). Once the user provides sequences in FASTA format, the algorithm calculates:

- *Solubility profiles.* To identify soluble fragments within each polypeptide chain, protein sequences are divided into elements and individual solubility propensities are calculated. Starting from the N-terminus of a protein, we use a sliding window of 21 amino acids that is moved one residue at a time until the C-terminus is reached. The solubility propensity profile of each fragment is calculated as defined in our previous publication (Agostini *et al.*, 2012).
- *Sequence susceptibility.* For each sequence analyzed, the algorithm computes the effect of single amino acid mutations at different positions. This approach is particularly useful to identify regions susceptible to solubility change upon mutation. All variants are reported along with their scores, which provides a basis to engineer protein sequences and test hypotheses such as the occurrence of specific mutations in pathology.
- *Solubility score.* The solubility profile represents a unique *signature* containing information on all fragments arranged in sequential order. In our approach, the profile is used to estimate solubility upon expression in the *E.coli* system. As sequences have different lengths, we exploit a method based on Fourier’s transform (Bellucci *et al.*, 2011; Tartaglia *et al.*, 2007) that allows comparison of polypeptide chains with different sizes. Using 100 Fourier’s coefficients, we trained an algorithm that has the same architecture developed for the analysis of protein expression levels in *E.coli* [i.e. neural network approach (Tartaglia *et al.*, 2009)].
- *Reliability score.* The webserver provides a confidence score based on statistical analysis of both training and testing sets (i.e. sequence range used to validate the method; see Supplementary Material).

All the aforementioned analyses are performed for each submitted protein set if the number of entries is <500. Because of the intense CPU usage, sequence susceptibility scores are not computed for datasets >500 entries.

## 3 PERFORMANCES

**Fig. 1.**
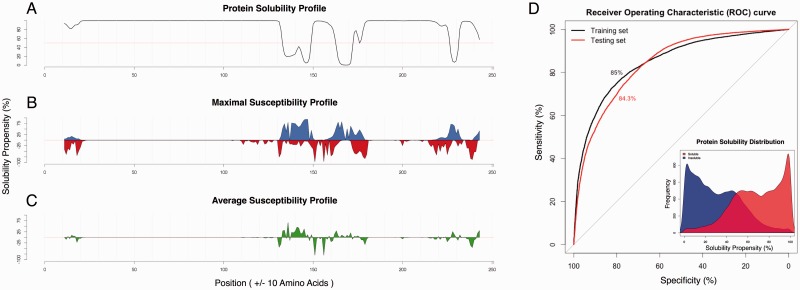
Human Prion Solubility and *cc*SOL Performances. (**A**) Starting from the N-terminus, *cc*SOL computes the solubility profile using a sliding window moved toward the C-terminus. *cc*SOL identifies the fragment 130–170 as the most insoluble within the C-terminus of human PrP (region 231–253 is not present in the mature form of the protein). (**B, C**) Maximal and average susceptibility upon single-point mutation. (**D**) We trained on the Target Track set (AUROC = 85.5%) and tested on *E.coli* [AUROC = 93.3%; (Niwa *et al.*, 2009)], SOLpro [AUROC = 85.7%; (Magnan *et al.*, 2009)] and PROSO II [AUROC = 82.9%; (Smialowski *et al.*, 2012)] proteins. Inset: overall score distribution for soluble (red) and insoluble (blue) proteins

Expression of human prion (PrP) in *E.coli* is particularly difficult, as the protein accumulates in inactive aggregates (Baneyx and Mujacic, 2004). *cc*SOL *omics* correctly predicts that PrP is insoluble and identifies the fragment 130–170 as the least soluble (Fig. 1A–C) together with region 231–253 (not present in the mature form). This finding is very well in agreement with what has been previously reported in literature (Tartaglia *et al.*, 2005, 2008). Moreover, the analysis of susceptible fragments identifies a number of experimentally validated mutations (e.g. G131V, S132I, R148H, V176I and D178N) associated with lower solubility and located in the region promoting PrP aggregation (Corsaro *et al.*, 2012) [see Supplementary Material]. As for the large-scale performances of *cc*SOL *omics*, we used a 10-fold cross-validation on Target Track [total of 36 990 entries with 30% redundancy (Fu *et al.*, 2012)] and observed 79% accuracy in discriminating between soluble and insoluble proteins. Furthermore, we tested the algorithm on three independent datasets containing protein expression data [total of 31 760 entries taken from *E.coli* (Niwa *et al.*, 2009), SOLpro (Magnan *et al.*, 2009) and PROSO II (Smialowski *et al.*, 2012)] and found 74% accuracy (Fig. 1D; see also Supplementary Material).

## 4 CONCLUSIONS

The *cc*SOL *omics* algorithm shows excellent performances in predicting solubility of endogenous and heterologous genes in *E.coli*. We hope that the webserver will be useful for biotechnological purposes, as it could be for instance used to design fusion tags for soluble expression. Although accurate, our calculations are based on sequence features, and integration with structural characteristics will dramatically increase the predictive power. We plan to combine *cc*SOL *omics* with information on chaperone (Tartaglia *et al.*, 2010) and RNA (Bellucci *et al.*, 2011; Choi *et al.*, 2009) interactions, as these molecules greatly contribute to the solubility of protein products (Cirillo *et al.*, 2014; Zanzoni *et al.*, 2013).
